# Papilledema and venous stasis in patients with cerebral venous and sinus thrombosis

**DOI:** 10.1186/s12883-023-03228-0

**Published:** 2023-04-28

**Authors:** Min-Gyu Park, Jieun Roh, Sung-Ho Ahn, Kyung-Pil Park, Seung Kug Baik

**Affiliations:** 1 Department of Neurology, Pusan National University Yangsan Hospital, Research Institute for Convergence of Biomedical Science and Technology, Pusan National University School of Medicine, 20 Geumo-Ro, Mulgeum, 50612 Yangsan, Republic of Korea; 2 Department of Radiology, Pusan National University Yangsan Hospital, Pusan National University School of Medicine, Yangsan, Republic of Korea

**Keywords:** Cerebral venous thrombosis, Papilledema, Intracranial pressure, Susceptibility-weighted imaging

## Abstract

**Background:**

Cerebral venous and sinus thrombosis (CVST) can cause increased intracranial pressure, often leading to papilledema. In this study, we investigated the association between papilledema and venous stasis on susceptibility weighted imaging (SWI) in CVST.

**Methods:**

Patients with CVST between 2008 and 2020 were reviewed. Patients without fundoscopic examination or SWI were excluded in this study. Venous stasis was evaluated and scored for each cerebral hemisphere: each hemisphere was divided into 5 regions according to the venous drainage territories (superior sagittal sinus, Sylvian veins, transverse sinus and vein of Labbé, deep cerebral veins, and medullary veins) and 1 point was added if venous prominence was confirmed in one territory on SWI. The venous stasis score on SWI between cerebral hemispheres with and without papilledema was compared.

**Results:**

Eight of 19 patients with CVST were excluded because of the absence of fundoscopic examination or SWI. Eleven patients (26.5 ± 2.1 years) were included in this study. Papilledema was identified in 6 patients: bilateral papilledema in 4 patients and unilateral papilledema in 2 patients. The venous stasis score on SWI was significantly higher (*P* = 0.013) in the hemispheres with papilledema (median, 4.0; 95% CI, 3.038–4.562) than in the hemispheres without papilledema (median, 2.5; 95% CI, 0.695–2.805).

**Conclusions:**

This study shows that higher score of venous stasis on SWI is associated with papilledema. Therefore, the venous stasis on SWI may be an imaging surrogate marker of increased intracranial pressure in patients with CVST.

**Supplementary Information:**

The online version contains supplementary material available at 10.1186/s12883-023-03228-0.

## Introduction

Cerebral venous and sinus thrombosis (CVST) is one of the main causes of papilledema [1]. Papilledema is a well-known marker of increased intracranial pressure (ICP). Without treatment, papilledema can cause permanent vision loss. Because papilledema in CVST can be asymptomatic and the majority of clinicians are not familiar with the use of ophthalmoscope, detection of papilledema can be delayed [2, 3]. Such diagnostic delays may be associated with an increased risk of visual deficit [4]. Therefore, it is important to identify papilledema in patients with CVST as early as possible [5].

In CVST, ICP can be increased due to venous stasis by venous outflow obstruction [6]. Although venous collateral can contribute to distributing venous stasis, this does not have a significant effect on the prognosis in patients with CVST [7]. If there were an imaging tool to assess for venous stasis in CVST, it would be available as an imaging surrogate marker for increased ICP in CVST. However, it is difficult to evaluate venous stasis with conventional magnetic resonance imaging (MRI) such as diffusion-weighed imaging, perfusion-weighted imaging, or magnetic resonance venography (MRV).

Venous stasis caused by CVST can lead to increased concentration of deoxyhemoglobin in draining veins [8]. Susceptibility-weighted imaging (SWI) shows cerebral venous structures very well by using magnetic susceptibility effects from paramagnetic deoxyhemoglobin [9]. For such reason, SWI can visualize venous stasis in CVST by exaggerating draining veins with higher deoxyhemoglobin [10–12]. Therefore, we hypothesized that venous stasis assessed by SWI could be an imaging surrogate marker of increased ICP in CVST. To test this hypothesis, we investigated whether venous stasis score on SWI is related to papilledema in patients with CVST.

## Methods

### Patients

We conducted a retrospective study on patients with CVST admitted to our institute between 2008 and 2020. CVST was diagnosed using conventional brain MRI, MRA, and MRV. Patients with the following conditions were excluded: (1) no fundoscopic examination, (2) no SWI, (3) venous infarction with mass effect or hemorrhage with mass effect at the time of diagnosis. Clinical symptoms, neurological signs, risk factors of CVST, and location of thrombus, and ophthalmologic evaluations including fundoscopic examination, visual acuity test, Humphrey visual field test, and intraocular pressure test were reviewed in all patients. This study adhered to the tenets of the Declaration of Helsinki which was approved by the Ethics Committee of Pusan National University Yangsan Hospital (Institutional Review Board number: 05–2021-167). Written informed consent was not required due to the retrospective nature of this study.

### Neuroimaging and image analysis

Patients were imaged with 3 T MRI systems (Verio or Skyra; Siemens, Germany) using commercially available software (syngo MR D13; Siemens Healthcare, Germany). Imaging protocols were included T2-weighted imaging, T1-weighted imaging, enhanced T1-weighted imaging, fluid-attenuated inversion recovery, diffusion-weighed imaging, SWI, and MRV or contrast MRV. Minimum intensity projections of SWI images with thickness of 16 mm were used to evaluate venous stasis.

**Fig. 1:**
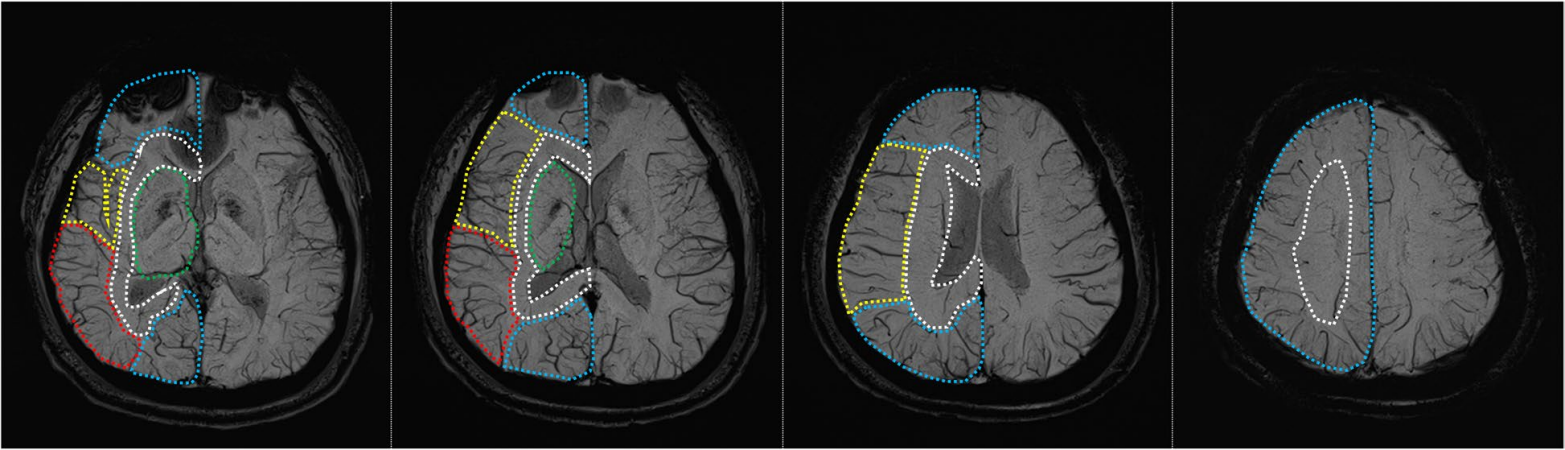
Regions on susceptibility-weighted imaging according to the venous territory: the region that drains into the superior sagittal sinus (blue); the region that drains into the deep cerebral veins (green), and medullary veins (white); the region that drains into the Sylvian veins (yellow); the region that drains into the transverse sinus and vein of Labbé (red)

**Fig. 2:**
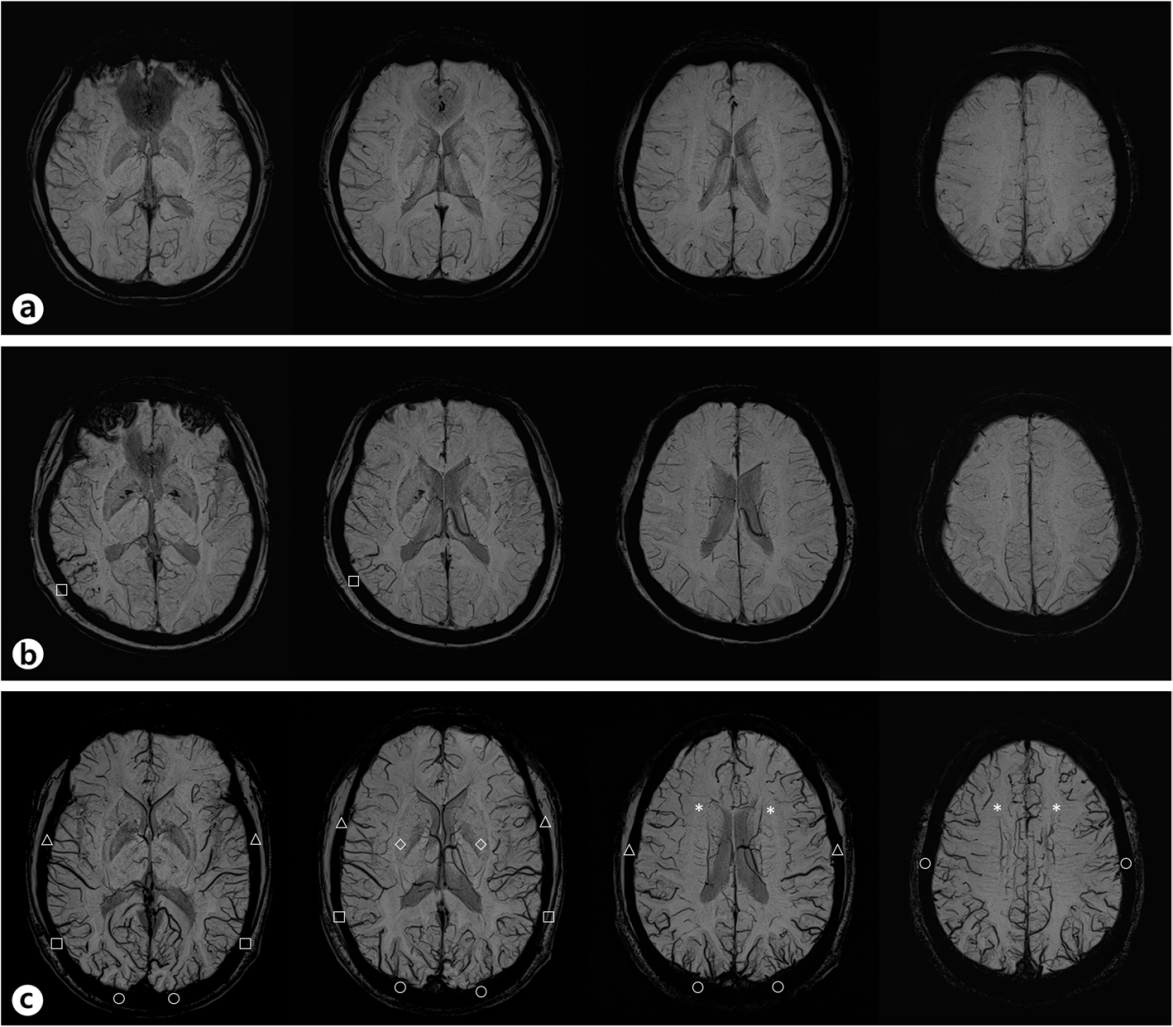
Examples of scoring the venous stasis on SWI. **a** The SWI images of the upper row are obtained from healthy control. **b** The SWI images of the middle row shows the venous stasis score of 1 in the right hemisphere because of exaggerated veins in the one territory of the transverse sinus and vein of Labbé (□). **c** The SWI images of the lower row demonstrates the venous stasis score of 5 in each of the two hemispheres because of exaggerated veins in the five territories (□, △, ◇, ○, *) of draining veins

The severity of venous stasis on SWI was scored separately for each cerebral hemisphere using the method of Sato et al.: each hemisphere was divided into 5 regions according to the venous drainage territories (superior sagittal sinus, Sylvian veins, transverse sinus and vein of Labbé, deep cerebral veins, and medullary veins) and 1 point was added if venous prominence was confirmed in one territory on SWI compared with SWI of healthy adults of the same age group (Fig. 1) [11]. SWI were reviewed independently by two readers who were blinded to the clinical data, each with 11 years and 9 years of experience in stroke neurology. The discordance of score between two reviewers was decided by consultation with a neuroradiologist with 26 years of experience. Representative cases are shown in Fig. 2.

### Statistical analysis

Inter-observer variability of the venous stasis score on SWI was analyzed by kappa statistics. The venous stasis scores on SWI were compared between hemispheres with and without papilledema by the Mann–Whitney U test. The time interval from first symptoms to diagnosis were compared between patients with and without papilledema by the Mann–Whitney U test. Receiver operating characteristic (ROC) curves were plotted for the venous stasis score on SWI for prediction papilledema, and optimal cut-off values were determined. We analyzed the data using Statistical Package for Social Sciences software for windows (Version 21; IBM, Armonk, New York). Statistical significance was defined as *P* < 0.05.

## Results

A total of 19 patients were diagnosed with CVST between 2008 and 2020. Eight of them were excluded due to absence of fundoscopic examination or SWI. Eleven patients (4 females; mean age 26.5 ± 2.1 years) were finally included in this study. Clinical characteristics of patients are shown in Table 1. Headache was the most frequent initial symptom (11 in 11 patients, 100%) in the patients. The causes of CVST were as follows: hyperhomocysteinemia (18.2%), antiphospholipid antibody syndrome (9.0%), nephrotic syndrome (9.0%), systemic lupus erythematous (9.0%), cancer (9.0%), primary myelofibrosis (9.0%), and no cause (46.4%). Intraocular pressure (range 8–16 mmHg) was normal in all patients. Mean visual acuity was 1.05 ± 0.21. Papilledema was identified in 6 patients (54.5%): bilateral papilledema in 4 patients (36.4%) and unilateral papilledema in 2 patients (18.2%). There was no visual filed defect in all patients on confrontational visual field exam, but Humphrey visual field testing showed an enlarged blind spot in left eye of one patient with bilateral papilledema. The mean time interval from first symptoms to diagnosis was 12.5 ± 12.0 days: 3.5 ± 0.7 days in 6 patients with papilledema and 15 ± 3.5 days in 5 patients without papilledema (*P* = 0.028). The average time from CVST diagnosis to papilledema documentation was 4.5 ± 0.7 days. Ten patients (90.9%) were treated with anticoagulation (bridging heparin to warfarin in 5 patients and warfarin without bridging heparin in 5 patients) and one patient (9.1%) was treated with aspirin as an alternative to anticoagulation because of the patient's concern about bleeding risk of anticoagulation. Four patients (36.4%) were treated with mannitol. The median follow-up period was 60 months (range 7–113 months). Complete recanalization was observed in 7 patients (63.6%) and partial recanalization in 2 patients (18.2%) at follow-up. Two patients (18.2%) had no recanalization. Papilledema was improved in all patients with papilledema within their follow-up period. The mean time to papilledema resolution was 47.4 ± 40.5 days.

**Table 1:** Clinical characteristics of subjects

Characteristics	All patients (*n* = 11)
Age (years), mean ± SD	26.5 ± 2.1
Female, n (%)	4 (36.4)
Initial symptoms, n (%)	
Headache	11 (100)
Seizure	4 (36.4)
Diplopia	1 (9.0)
Tinnitus	1 (9.0)
Papilledema, n (%)	6 (54.5)
Bilateral	4 (36.4)
Unilateral	2 (18.2)
Time interval from first symptom to diagnosis (days), mean ± SD	12.5 ± 12.0
Cause, n (%)	
Hyperhomocysteinemia related with MTHFR 677 T/T	2 (18.2)
Antiphospholipid antibody syndrome	1 (9.0)
Nephrotic syndrome	1 (9.0)
Systemic lupus erythematous	1 (9.0)
Cancer	1 (9.0)
Primary myelofibrosis	1 (9.0)
No definite cause	4 (46.4)

*SD* standard deviation*, MTHFR* Methylenetetrahydrofolate reductase

**Fig. 3:**
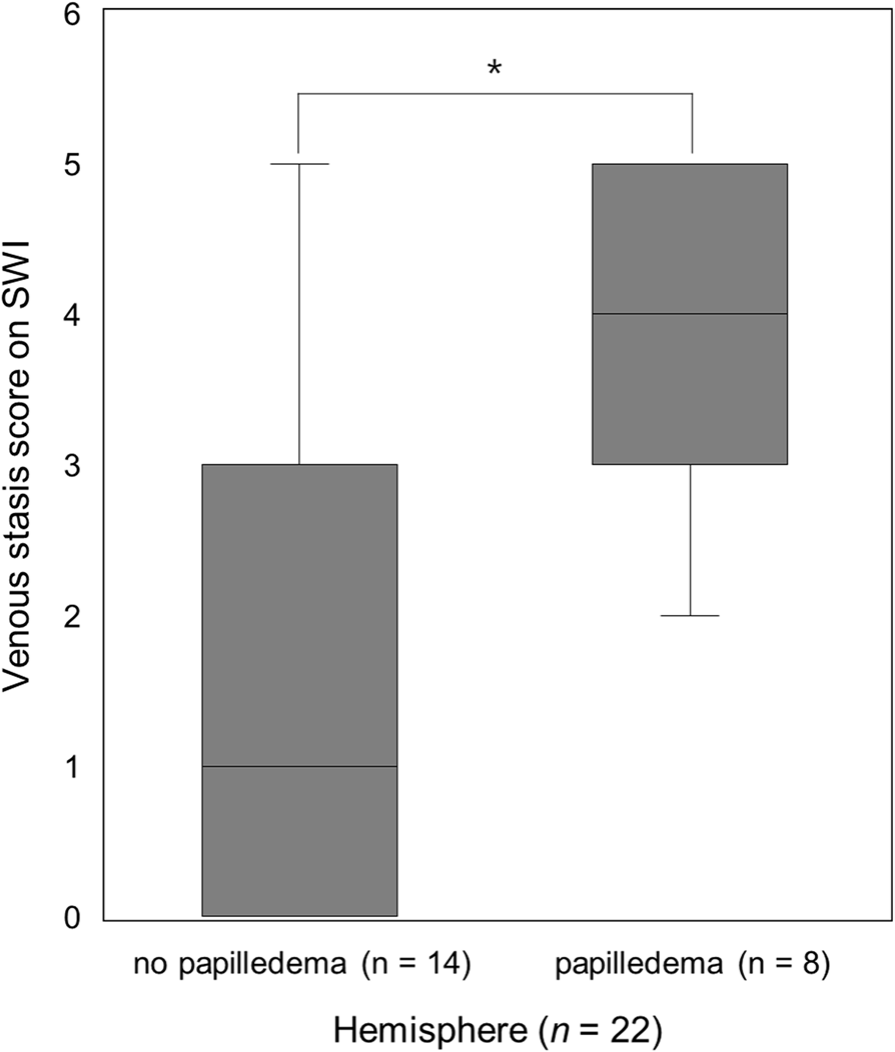
Comparison of the venous stasis score on SWI between hemispheres with and without papilledema. The scores of venous stasis on SWI are significantly higher in the cerebral hemisphere with papilledema (median, 4; 95% CI, 3.04–4.56) than in the cerebral hemisphere without papilledema (median, 2.5; 95% CI, 0.70–2.81). * indicates *P* = 0.013

The venous stasis score on SWI and sites of venous occlusion are summarized in Table 2. The transverse sinus was the most common location of occlusion (8 patients, 72.7%). The venous stasis score on SWI showed a good agreement between the 2 evaluators (k = 0.813, *P* < 0.001). The scores of venous stasis on SWI were significantly higher (*P* = 0.013) in the cerebral hemisphere with papilledema (median, 4.0; 95% CI, 3.04–4.56) than in the cerebral hemisphere without papilledema (median, 2.5; 95% CI, 0.70–2.81) (Fig. 3). We performed a ROC analysis for venous stasis score on SWI, headache, and nausea/vomiting to predict papilledema in CVST patients (Figure S1 in the Supplement). The area under the ROC curve of venous stasis score on SWI was 0.808 (*P* = 0.001; 95% CI, 0.620–0.996). The best cut-off value for the venous stasis score on SWI was 3.5 (sensitivity of 60% and specificity of 83.3%). The areas under the ROC curve of headache and nausea/vomiting were 0.500 (*P* = 1.000; 95% CI, 0.252–0.748) and 0.617 (*P* = 0.356; 95% CI, 0.374–0.860).

**Table 2:** The venous stasis score on SWI in all patients

Patients	Venous stasis score in each venous territory (R/L)					Total score of venous stasis in each hemisphere (R/L)	Site of occlusion	Papilledema
	SSS	SV	TS/VL	DCV	MV			
1	1/1	1/1	1/1	0/0	0/0	3/3	SSS, CV	Bilateral
2	1/1	1/0	1/1	1/1	1/1	5/4	right TS, SSS	Bilateral
3	1/1	1/1	1/1	1/1	1/1	5/5	right TS, SSS, CV	Bilateral
4	1/1	1/0	1/0	0/0	1/1	4/2	SSS, bilateral TS	Bilateral
5	1/0	0/0	0/0	0/0	1/0	2/0	CV	Right
6	1/1	1/1	1/1	1/1	1/1	5/5	SSS	Right
7	1/1	0/0	0/0	0/0	1/0	2/1	SSS, CV	None
8	0/0	0/0	0/0	0/0	0/0	0/0	right TS, right SS	None
9	0/0	0/0	1/0	0/0	0/0	1/0	right JV, right SS, right TS	None
10	1/1	0/1	0/1	1/1	1/1	3/5	left TS, left SS, left JV	None
11	0/1	0/0	0/1	0/0	1/1	1/3	left TS, left SS	None

*SSS* superior sagittal sinus, *SV* Sylvian veins, *TS* transverse sinus, *VL* vein on Labbé, *DCV* deep cerebral veins, *MV* medullary veins, *CV* cortical vein, *TS* transverse sinus, *SS* sigmoid sinus, *JV* jugular vein

## Discussion

In this study, CVST patients with higher score of venous stasis on SWI were more likely to have papilledema. This result supports our hypothesis that venous stasis assessed by SWI could be a neuroimaging surrogate marker of ICP in CVST. One previously published study suggested that venous stasis evaluated by SWI can help predict cerebrovascular complications in CVST [11]. Venous stasis in CVST is closely related to increased venous pressure due to venous outflow obstruction [6]. Increased venous pressure directly increases venular and capillary pressure or indirectly affect them by elevating ICP, which contribute to various clinical problems such as headache, seizure, focal neurological deficits, and papilledema [5, 6]. In our study, 54.5% of CVST patients had papilledema at the time of diagnosis. In the study of Liu et al., 40% of CVST patients with papilledema suffered permanent visual damage, but all patients in our study recovered without permanent visual damage [1]. Better outcomes of our study might be because the mean time from CVST diagnosis to detection of papilledema was shorter than that in their study, 4.5 days versus 29 days. According to the experimental model of papilledema, in the majority of cases, when ICP elevated, optic disc edema appeared within 7 day; however, there were some cases when optic disc edema appeared within 24 h at the earliest after ICP elevated [13]. Therefore, even if there is no symptoms of papilledema at the time of diagnosis of CVST, fundus examination is necessary to detect the early sign of papilledema in all CVST patients, especially in those with significant venous stasis on SWI.

One meta-analysis showed that MRV has excellent diagnostic performance in confirming CVST [14]. In particular, contrast MRV increased the specificity in diagnosis of CSVT comparable to digital subtraction angiography [14]. However, neither time-of-flight MRV nor contrast MRV is sufficient for visualizing fine venous structures such as small cortical and medullary veins due to the limitations of spatial resolution [15–17]. Therefore, MRV is not a suitable imaging technique to evaluate the venous stasis in fine cortical and medullary veins. The venous stasis increases the amount of deoxyhemoglobin in draining veins [10]. Each human red blood cell (RBC) contains approximately 270 million hemoglobin molecules [18]. A fully deoxygenated hemoglobin has four unpaired electrons. Therefore, if one RBC is completely unsaturated, it will have approximately 1,080 million unpaired electrons, which are sources of a strong paramagnetic property [9]. The paramagnetic property of deoxyhemoglobin makes susceptibility difference from the background brain tissue. Therefore, the greater the amount of deoxyhemoglobin in the veins, the greater the difference in susceptibility between the veins and the surrounding tissue. In that aspect, SWI is a useful imaging tool for visualizing the venous stasis in fine cortical and medullary veins because SWI uses deoxyhemoglobin as an intrinsic contrast agent [9]. SWI also can show the prominent cortical and medullary veins in acute cerebral infarction due to arterial occlusion, but its mechanism is slightly different from that of CVST; in acute cerebral infarction due to arterial occlusion, an increase in the ratio of intravenous deoxyhemoglobin to oxyhemoglobin is the mechanism, whereas the mechanism of prominent cortical and medullary veins on SWI in CVST is the increase in the amount of intravenous hemoglobin itself due to venous stasis [19, 20].

In this study, unilateral papilledema was detected in 2 patients. Unilateral papilledema due to increased ICP is an atypical presentation, but it can be found in the literature [21–23]. Although the exact mechanism of unilateral papilledema caused by increased ICP has not been revealed yet, the mechanisms can be summarized as the following three: 1) an anomaly in the orbital optic nerve sheaths; 2) a difference in the lamina cribrosa between the two optic discs that results in reduced transmission of the intracranial pressure to the optic nerve in the scleral canal; 3) an abnormality in the venous sinuses [21–23]. In this study, we did not evaluate abnormality of the optical nerve sheath and lamina cribrosa in the included patients. However, we confirmed that 2 patients with unilateral papilledema have ipsilateral transverse sinus hypoplasia. Recently published small case series study showed that unilateral transverse sinus hypoplasia may be associated with CVST [24]. Even so, it is difficult to conclude that unilateral transverse sinus hypoplasia is directly related to CVST because the prevalence of unilateral transverse sinus hypoplasia is known to be about 30% in the general population [25]. However, it is likely that unilateral transverse sinus hypoplasia provides a narrow passage to disturb the venous outflow and aggravate the venous stasis in the ipsilateral hemisphere with CVST.

There are several limitations to our study. First, it is a retrospective study with small sample size, which is vulnerable to selective bias and weak conclusions. However, small case series of a rare disease can provide important information to clinicians who have less opportunities to experience that disease because of its rarity [26]. Therefore, considering a rarity of CVST, the results of our study can be helpful to clinicians in understanding the pathogenesis of CVST. Nevertheless, the small number of cases is not enough to conclude our hypothesis, and further research with a large number of cases is essential to confirm the results of this study. Second, we were not able to evaluate the association between the venous stasis on SWI and cerebrospinal fluid (CSF) opening pressure. In 5 patients, anticoagulation therapy had already started, so lumbar puncture was not performed in them. CSF opening pressure is known to be a surrogate measurement of ICP. However, it is also true that CSF opening pressure can show variability among normal individuals by age and body mass index [27–29]. Even if CSF opening pressure was measured in all patients in this study, it would have been difficult to correct for the effects of age and BMI due to the small number of subjects. The final limitation is that there is no normal reference for venous prominence on SWI in general population. Despite the limitation, to evaluate the venous stasis on SWI in this study, we had to compare SWI of the included patients with SWI of healthy adults. The reason is that venous stasis can develop in bilateral hemispheres in patient with superior sagittal sinus thrombosis; in this situation, the venous stasis cannot be evaluated by comparing between bilateral hemispheres [10].

## Conclusions

This study shows that higher score of venous stasis on SWI is associated with papilledema. Therefore, the venous stasis on SWI may be an imaging surrogate marker of increased intracranial pressure in patients with CVST. Further studies are needed to confirm our results.

## Supplementary Information

**Additional file 1:** **FigureS1.** Receiver-operatingcharacteristic (ROC) curves for venous stasis score on SWI, headache, and nausea/vomiting.For venous stasis score on SWI, the area under the curve was 0.808, withsensitivity of 60% and specificity of 83.3% (*P* = 0.001; 95% CI, 0.620–0.996). Theareas under the ROC curve of headache and nausea/vomiting were 0.500 (*P* = 1.000; 95% CI, 0.252–0.748) and0.617 (*P* = 0.356; 95% CI,0.374–0.860).

## Data Availability

The data sets used and/or analyzed during the current study are available from the corresponding author on reasonable request.

## Abbreviations

CVST: Cerebral venous and sinus thrombosis
ICP: Intracranial pressure
MRI: Magnetic resonance imaging
MRV: Magnetic resonance venography
SWI: Susceptibility-weighted imaging
ROC: Receiver operating characteristic
RBC: Red blood cell
CSF: Cerebrospinal fluid
