# Supplementary material for: Papilledema and venous stasis in patients with cerebral venous and sinus thrombosis
Source: BMC Neurol. 2023 Apr 28;23:175. doi: 10.1186/s12883-023-03228-0 (PMC10148469; doi:10.1186/s12883-023-03228-0)
Supplement: Supplementary file 1 — Additional file 1: FigureS1. Receiver-operatingcharacteristic (ROC) curves for venous stasis score on SWI, headache, and nausea/vomiting.For venous stasis score on SWI, the area under the curve was 0.808, withsensitivity of 60% and specificity of 83.3% (P = 0.001; 95% CI, 0.620–0.996). Theareas under the ROC curve of headache and nausea/vomiting were 0.500 (P = 1.000; 95% CI, 0.252–0.748) and0.617 (P = 0.356; 95% CI,0.374–0.860). [file 12883_2023_3228_MOESM1_ESM.docx]

**Supplementary Online Content**

Figure S1. Receiver-operating characteristic (ROC) curves for venous stasis score on SWI, headache, and nausea/vomiting.


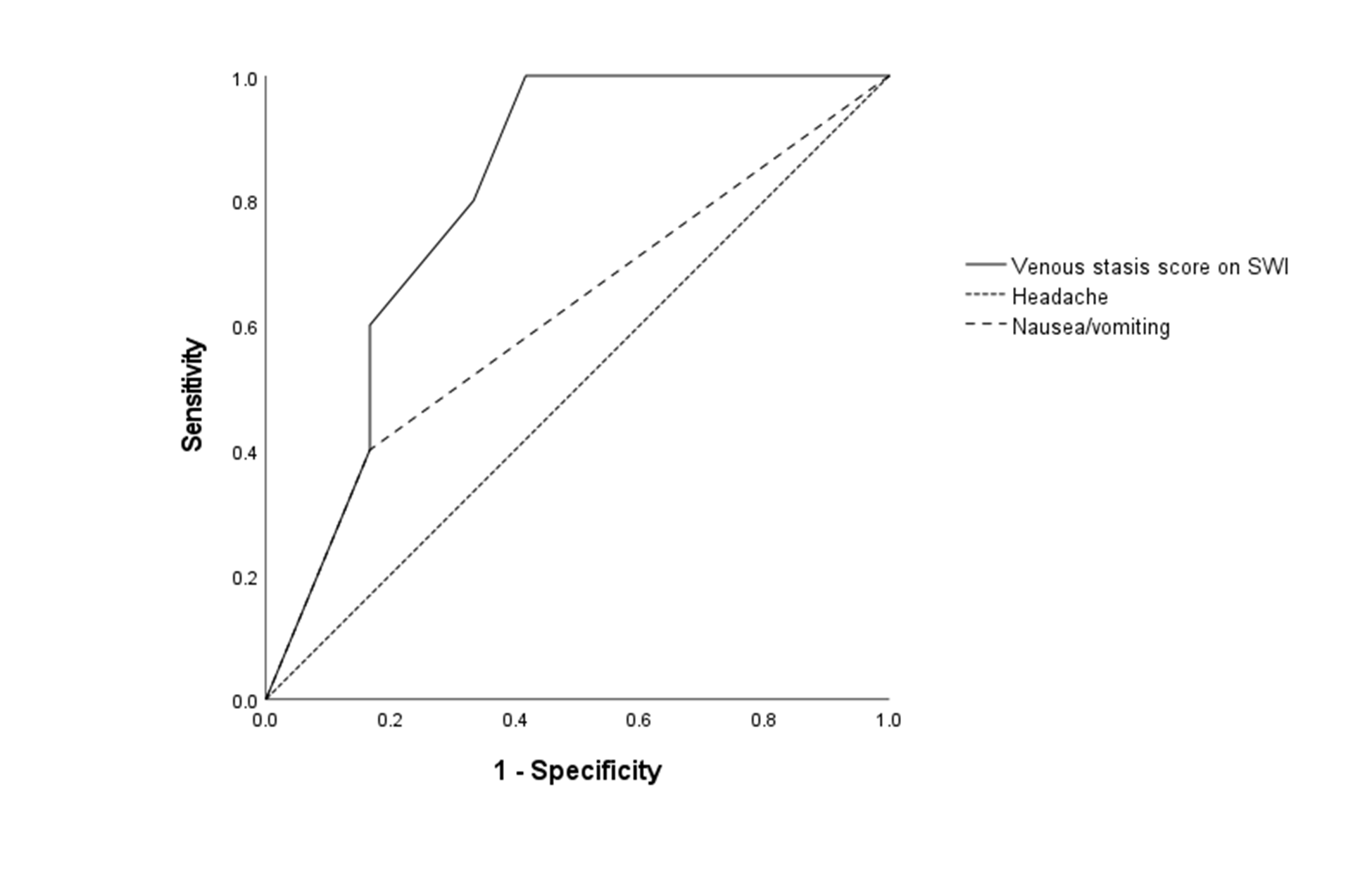


**Figure S1.** Receiver-operating characteristic (ROC) curves for venous stasis score on SWI, headache, and nausea/vomiting. For venous stasis score on SWI, the area under the curve was 0.808, with sensitivity of 60% and specificity of 83.3% (*P* = 0.001; 95% CI, 0.620–0.996). The areas under the ROC curve of headache and nausea/vomiting were 0.500 (*P* = 1.000; 95% CI, 0.252–0.748) and 0.617 (*P* = 0.356; 95% CI, 0.374–0.860).
